# Interatrial Conduction Block in Pediatric Patients with Ostium Secundum Atrial Septal Defect

**DOI:** 10.3390/jcm15051916

**Published:** 2026-03-03

**Authors:** Silvia Garibaldi, Fabiana Lucà, Francesca Valeria Contini, Alessandra Pizzuto, Gianluca Mirizzi, Massimiliano Cantinotti, Martina Nesti, Luca Panchetti, Umberto Startari, Marcello Piacenti, Nadia Assanta, Andrea Rossi, Federico Landra, Giuseppe Santoro

**Affiliations:** 1 Tuscany Region-National Research Council Foundation “G. Monasterio”, San Cataldo Hospital, 56124 Pisa, Italy; sgaribaldi@ftgm.it (S.G.); gmirizzi@ftgm.it (G.M.); lucapanchett@ftgm.it (L.P.);; 2 Cardiology Department, Grande Ospedale Metropolitano GOM, 89124 Reggio Calabria, Italy; 3 Department of Medical Biotechnologies, Division of Cardiology, University of Siena, 53100 Siena, Italy; fvcontini@gmail.com (F.V.C.); alepizzuto89@gmail.com (A.P.); cantinotti@ftgm.it (M.C.);; 4 Tuscany Region-National Research Council Foundation “G. Monasterio”, Heart Hospital “G. Pasquinucci”, 54100 Massa, Italy

**Keywords:** interatrial conduction, interatrial block, atrial septal defect, Bachmann’s bundle, percutaneous closure

## Abstract

**Background:** Atrial arrhythmias represent a frequent long-term complication in patients with atrial septal defects (ASDs). Interatrial block (IAB), reflecting delayed or impaired conduction across Bachmann’s bundle, has been proposed as an electrophysiological substrate predisposing to atrial arrhythmogenesis. However, evidence regarding its prevalence and clinical correlates in pediatric patients with ASD remains limited. The present study aimed to characterize interatrial conduction patterns and assess the occurrence of IAB in children with large secundum ASD undergoing percutaneous closure. **Methods:** Between January 2020 and March 2024, 37 consecutive pediatric patients (median age 6 years, range 5–11) with large ostium secundum ASD were included in a retrospective analysis of a prospectively maintained institutional database. Standard 12-lead electrocardiograms were recorded before and within 24 h after defect closure. P-wave morphology and duration were systematically analyzed, and IAB was classified according to the Bayés de Luna criteria. **Results:** The median Qp/Qs ratio was 1.69 (1.32–2.24), with a mean pulmonary artery pressure of 19 mmHg (17–22). IAB was identified in 24.3% of patients before the procedure, predominantly as first-degree IAB. Following device implantation, IAB prevalence (29.7%) and P-wave parameters remained unchanged, with no significant differences compared with baseline. No associations were observed between IAB and defect size, hemodynamic burden, or device characteristics, whereas anthropometric variables, including weight, height, and body surface area, showed a significant correlation with IAB occurrence. During a median follow-up of 199 days, no atrial arrhythmias were documented. **Conclusions:** In this pediatric cohort with large ASD, IAB was present in approximately one quarter of patients and appeared unrelated to anatomical or procedural factors, supporting the hypothesis of an underlying congenital conduction abnormality. Early recognition of IAB may therefore have implications for long-term arrhythmic risk stratification in this population.

## 1. Introduction

Interatrial electrical coupling is ensured by a complex network of preferential conduction pathways that allow rapid and coordinated activation of both atria. Among these, Bachmann’s bundle (BB) represents the principal and most efficient interatrial connection, providing fast conduction from the right atrium (RA) to the left atrium (LA) [1,2]. In addition to BB, alternative conduction routes have been described, including muscular bundles located along the inferior atrial surface near the coronary sinus, transseptal fibers traversing the fossa ovalis, and posterior interatrial connections adjacent to the right pulmonary veins [1,2,3,4,5,6]. The relative contribution of each pathway is variable, but their integrated function is essential to preserve atrial synchrony.

BB is characterized by a conduction velocity nearly twice that of the surrounding atrial myocardium, making it the dominant interatrial pathway under physiological conditions [7]. Normal atrial activation occurs within 110 ms, a temporal interval that corresponds to the duration of the P wave on the surface electrocardiogram. Progressive impairment of BB conduction gives rise to interatrial block (IAB), a condition systematically classified by Bayés de Luna and colleagues on the basis of characteristic electrocardiographic patterns [8,9]. IAB has been increasingly recognized as a marker of atrial electrical vulnerability and has been consistently associated with atrial fibrillation, atrial flutter, and other supraventricular tachyarrhythmias [10,11,12,13]. These arrhythmias carry substantial clinical and societal implications, including thromboembolic events, cognitive impairment, heart failure progression, and excess mortality [14,15].

Anatomical variability further modulates interatrial conduction. BB may be partially hypoplastic or even absent, and the number, caliber, and spatial distribution of alternative interatrial pathways differ markedly among individuals [16,17]. Such heterogeneity may underlie interindividual differences in susceptibility to conduction delay and atrial arrhythmogenesis, even in the absence of overt structural heart disease.

Ostium secundum atrial septal defect (ASD) is one of the most common congenital heart defects, with an estimated prevalence of approximately 1 per 1000 live births. Atrial arrhythmias represent a frequent long-term complication in this population, affecting up to 40% of patients during follow-up [18,19,20,21]. In unrepaired ASD, chronic left-to-right shunting leads to right-sided volume overload, atrial dilatation, and progressive structural remodeling. These changes are accompanied by electrical remodeling, including increased dispersion of atrial refractoriness, conduction slowing at the crista terminalis, and sinus node dysfunction, all of which contribute to arrhythmic vulnerability [22,23,24,25].

Beyond volume overload, atrial conduction in ASD is thought to be intrinsically altered by the disruption of septal conduction pathways [26]. Although transseptal fibers within the fossa ovalis have been anatomically described, their functional role in atrial activation remains incompletely defined, and no specific electrocardiographic signatures have been clearly attributed to their absence in ASD. Nevertheless, dysfunction of other preferential interatrial pathways, particularly BB, may play a relevant but underexplored role in determining arrhythmic risk in this setting.

Despite the established association between IAB and atrial arrhythmias in the general population, its prevalence, determinants, and clinical significance in patients with ASD, especially in the pediatric age group, have not been systematically investigated. Moreover, although percutaneous ASD closure effectively corrects the hemodynamic burden, in the present study, no early arrhythmic events were observed; however, the limited duration of follow-up does not allow conclusions regarding long-term electrophysiological outcomes.

The present study was therefore designed to evaluate interatrial conduction patterns and the occurrence of interatrial block in a pediatric cohort with large ostium secundum ASD undergoing percutaneous closure with different device types.

## 2. Methods

### 2.1. Study Design and Population

This study represents a retrospective analysis of a prospectively maintained institutional database conducted at the Pediatric Cardiology and Adult Congenital Heart Disease (ACHD) Unit of the Heart Hospital “G. Pasquinucci” (Massa, Italy). The study protocol received approval from the local Institutional Review Board, and written informed consent was obtained from all participants or from their parents or legal guardians, when applicable. All procedures conformed to the ethical standards of the Declaration of Helsinki.

The study population included pediatric patients with isolated, large ostium secundum atrial septal defect (ASD) who underwent percutaneous defect closure. Large ASD was defined by an ASD-to-weight ratio > 1.2 and/or an ASD-to-body surface area (BSA) ratio > 20 mm^2^/m^2^. Patients aged ≥18 years and those with relevant cardiac or extracardiac comorbidities were excluded. Demographic data, clinical history, hemodynamic parameters, electrocardiographic findings, procedural details, and follow-up information were prospectively recorded in a dedicated institutional database and retrospectively analyzed.

### 2.2. Percutaneous ASD Closure Procedure

All interventions were performed under general anesthesia with combined fluoroscopic and transesophageal echocardiographic (TEE) guidance. Defect sizing was carried out using either static or dynamic techniques, according to operator preference and anatomical characteristics. Static sizing was performed with the Amplatzer Sizing Balloon (Abbott, Plymouth, MN, USA), inflated until complete abolition of the interatrial shunt was confirmed by TEE, defining the so-called stop-flow diameter. Dynamic sizing was obtained using a pulling (“stretched”) technique with an off-label sizing balloon (Equalizer Occluding Balloon, Boston Scientific, Cork, Ireland).

Device diameter selection was based on the measured ASD size, choosing a prosthesis within 2 mm of the assessed defect diameter in accordance with manufacturer recommendations. Various self-centering occluder devices were employed. Although in routine clinical practice, the Amplatzer Septal Occluder and similar devices are preferentially used for medium-sized defects (ASD/weight ratio < 1; ASD/BSA < 20 mm^2^/m^2^), it should be emphasized that all patients included in the present study fulfilled predefined criteria for large ASD.

In contrast, the GORE^®^ Cardioform ASD Occluder (WL Gore & Associates, Flagstaff, AZ, USA) was selected for larger defects, particularly when the anticipated risk of electromechanical interaction or device-related erosion was considered higher. In such cases, the GORE^®^ Cardioform device was favored because of its greater softness and superior anatomical compliance compared with other self-centering occluders, as supported by available evidence [27,28,29,30,31].

### 2.3. Electrocardiographic Assessment

Electrocardiographic analysis was performed manually by a single experienced investigator who was blinded to patients’ clinical, procedural, and follow-up data. Standard 12-lead electrocardiograms (ECGs) were recorded at a paper speed of 25 mm/s and a calibration of 1 mV/cm within 24 h after device implantation. When available, pre-procedural ECGs were retrieved from the institutional electronic archive or obtained directly from patients’ families. All ECG tracings were subsequently digitized, allowing magnification of signal amplitude and paper speed without loss of waveform resolution.

P-wave analysis was conducted in the time domain in accordance with previously validated methodologies [32,33]. The following parameters were systematically assessed:

- **Total P-wave duration:** interval from the earliest onset of the P wave in any lead to the latest offset in any lead (ms).
- **Maximum P-wave duration:** longest P-wave duration measured across all leads (ms).
- **Minimum P-wave duration:** shortest P-wave duration measured across all leads (ms).
- **Isoelectric interval:** difference between total and maximum P-wave duration (ms).
- **P-wave dispersion:** difference between maximum and minimum P-wave duration (ms).
- **P-wave axis:** frontal plane P-wave axis.
- **Maximum P-wave amplitude:** the highest P-wave amplitude recorded in any lead (mV).
- **Minimum P-wave amplitude:** the lowest P-wave amplitude recorded in any lead (mV).
- P-wave amplitude in lead I: measured in mV.
- **P-wave area:** calculated as the product of half the P-wave duration and P-wave amplitude in lead II (ms·mV).
- **P-wave terminal force in lead V1:** calculated as the product of the duration and depth of the terminal negative component of the P wave in lead V1 (ms·mm).

**Figure 1:**
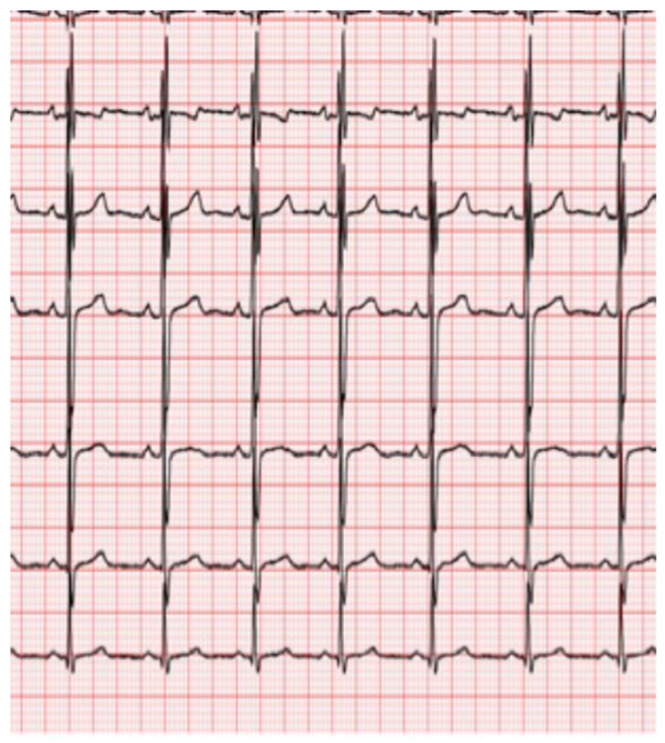
First-degree interatrial block. Note the bimodal configuration of the P wave in leads I, II, and aVF, with a biphasic P wave in lead V1.

**Figure 2:**
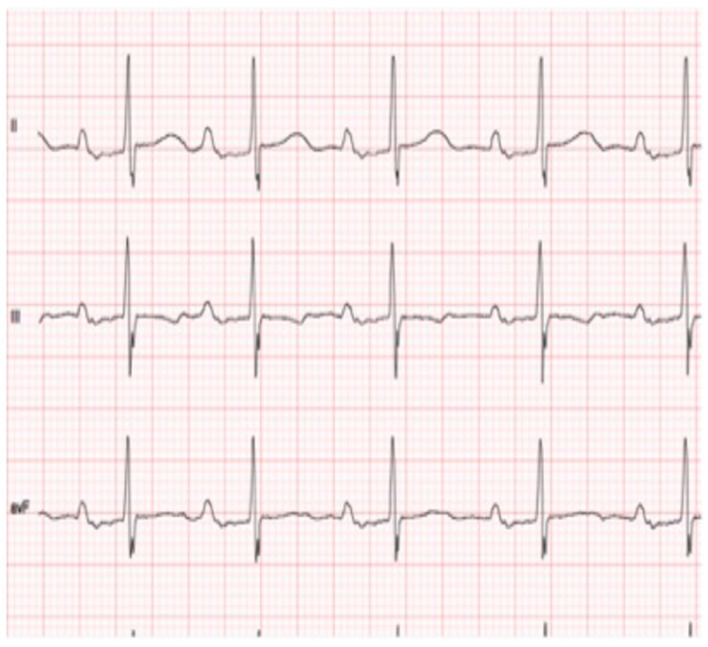
Third-degree interatrial block. Note the biphasic configuration in the inferior leads (II, III, and aVF).

**Figure 3:**
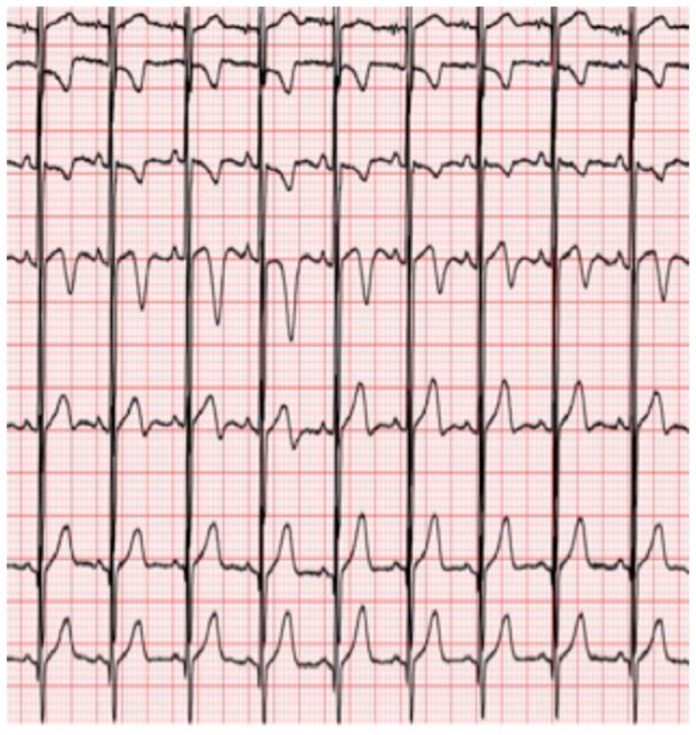
Atypical interatrial block pattern. Note the biphasic configuration of P wave in leads I, II, III, and aVF, even though not positive and without a biphasic configuration in lead V1.

Interatrial block (IAB) was defined according to established electrocardiographic criteria [34]. Although the 120 ms threshold was originally derived from adult populations, no age-specific diagnostic criteria for interatrial block have been validated in pediatric cohorts. Therefore, in the absence of pediatric reference standards, the established Bayés de Luna criteria were applied, integrating both duration and morphological features to minimize the risk of age-related misclassification. First-degree (partial) IAB was diagnosed in the presence of a P-wave duration ≥ 120 ms with a positive, typically bimodal morphology in leads I, II, III, and aVF, and a normal biphasic configuration in lead V1 (Figure 1). Second-degree (intermittent) IAB was defined by alternating patterns of partial and advanced block within the same ECG recording. Third-degree (advanced) IAB was identified by a P-wave duration ≥ 120 ms associated with a biphasic (positive–negative) morphology in the inferior leads (II, III, and aVF) (Figure 2). Atypical IAB patterns included a P-wave duration < 120 ms or the absence of biphasic morphology in all three inferior leads (Figure 3).

### 2.4. Follow-Up

Follow-up data were obtained from the most recent outpatient visit documented in the institutional electronic medical record. When follow-up evaluations were performed at external institutions, clinical information was collected through structured telephone interviews. The occurrence of atrial arrhythmias during follow-up was specifically assessed and recorded.

### 2.5. Statistical Analysis

Continuous variables are presented as mean ± standard deviation or as median with interquartile range, depending on their distribution. Normality of continuous data was assessed using the Kolmogorov–Smirnov test. Categorical variables are expressed as absolute frequencies and percentages. Between-group comparisons were performed using the unpaired Student’s *t*-test for normally distributed variables and the Mann–Whitney U test for variables with non-normal distribution. The χ^2^ test was used to compare categorical variables.

Given the relatively small sample size, regression analyses were deliberately limited to univariable and bivariable models in order to reduce the risk of model overfitting. These analyses were performed to explore associations between baseline clinical and anthropometric characteristics and the presence of interatrial block, and results are reported as odds ratios with corresponding confidence intervals. No multivariable models were constructed because of the limited number of outcome events.

All statistical tests were two-sided, and a *p* value < 0.05 was considered statistically significant. Statistical analyses were carried out using SPSS software, version 26 (IBM Corp., Chicago, IL, USA).

## 3. Results

### 3.1. Baseline Characteristics

Between January 2020 and March 2024, a total of 269 patients with ostium secundum atrial septal defect underwent percutaneous closure at our institution. Of these, 37 pediatric patients met the predefined inclusion criteria and were included in the present analysis. Baseline demographic, anatomical, and hemodynamic characteristics of the study population are summarized in Table 1.

When patients were stratified according to the presence or absence of interatrial block (IAB), significant differences emerged with respect to anthropometric parameters. Patients with IAB exhibited higher body weight [46 (24–56) vs. 21 (16–28) kg; *p* = 0.017], greater height [159 (123–169) vs. 115 (106–129) cm; *p* = 0.019], and larger body surface area (BSA) [1.4 (0.9–1.6) vs. 0.8 (0.7–1.0) m^2^; *p* = 0.024] compared with those without IAB. In contrast, absolute ASD diameter did not differ significantly between groups, whereas the ASD diameter-to-weight ratio was significantly lower in patients with IAB [0.7 (0.4–0.8) vs. 0.9 (0.8–1.2) mm/kg; *p* = 0.027]. None of the enrolled patients had a documented history of atrial arrhythmias prior to the procedure.

### 3.2. Electrocardiographic Findings

No significant differences were observed between pre-procedural and post-procedural P-wave parameters across the study population (*p* > 0.05 for all comparisons). The prevalence of IAB of any degree before ASD closure was 24.3%, which did not differ significantly from the post-procedural prevalence of 29.7% (*p* = 0.734). First-degree IAB represented the predominant electrocardiographic pattern, accounting for 88.8% of cases.

Following percutaneous ASD closure, three patients (8.1%) developed new-onset first-degree IAB, whereas resolution of pre-existing first-degree IAB was observed in one patient (2.7%). No progression from partial to advanced IAB was documented in patients with pre-procedural conduction abnormalities. Detailed electrocardiographic measurements and P-wave characteristics are reported in Table 2 and Table 3.

### 3.3. Association with Interatrial Block

Univariable logistic regression analysis did not identify significant associations between IAB occurrence and anatomical or hemodynamic parameters, nor with the type of ASD closure device used (*p* > 0.05 for all), although a nonsignificant trend was observed for ASD diameter. In contrast, anthropometric variables, including body weight, height, and BSA, were significantly associated with the presence of IAB (*p* < 0.05). Age showed a nonsignificant trend toward association. These findings suggest that IAB occurrence may increase with somatic growth at the time of ASD closure; however, the influence of timing of repair could not be formally assessed in this cohort.

To account for body size, a bivariable logistic regression model including ASD diameter and BSA was constructed; however, no significant association between these variables and IAB occurrence was observed. Complete results of the univariable and bivariable regression analyses are presented in Table 4.

### 3.4. Follow-Up

The median duration of follow-up was 199 days (range 94–377 days). During this period, no atrial arrhythmic events were recorded in the study population.

## 4. Discussion

In the present study, interatrial block (IAB) was identified in approximately one quarter of pediatric patients with large ostium secundum atrial septal defects (ASDs). Importantly, the presence of IAB was not associated with defect size, hemodynamic burden, or the percutaneous closure procedure itself. These findings suggest that interatrial conduction abnormalities may represent an intrinsic substrate in a relevant proportion of children with ASD, warranting careful attention because of their potential implications for long-term arrhythmic risk.

Within our cohort, IAB was predominantly expressed as a first-degree block. Patients with IAB differed significantly from those without IAB in terms of anthropometric parameters, including body weight, height, and body surface area, whereas the anatomical and hemodynamic characteristics of the defect were comparable between groups. This observation may suggest that a pre-existing interatrial conduction vulnerability becomes more evident with somatic growth and atrial maturation. Alternatively, progressive atrial remodeling related to chronic volume overload cannot be excluded. The lack of association with defect size or hemodynamic burden, however, argues against a purely load-dependent mechanism. The association with body size may also reflect the timing of ASD closure, as older children undergoing later repair could have been exposed for a longer period to right-sided volume overload and atrial remodeling. Although no significant relationship with defect size or hemodynamic burden was observed in this cohort, the potential influence of age at repair cannot be excluded. Given the limited sample size, stratified analyses by timing of intervention were not feasible; this warrants further investigation in larger studies.

The prevalence of IAB observed in our pediatric population was lower than that reported in a large cohort of unselected hospitalized adults (38.8%) [35]. Methodological differences may partly account for this discrepancy. In the study by Frisella et al., IAB detection was based on P-wave analysis across all ECG leads, rather than on lead II alone as traditionally proposed, resulting in a substantially higher detection rate. In the present study, IAB classification strictly followed the criteria established by Bayés de Luna et al., which may have limited the identification of non-classical or borderline conduction abnormalities.

Although disruption of septal conduction pathways might be expected in patients with ostium secundum ASD, our findings suggest that conduction through Bachmann’s bundle (BB) may also be frequently impaired. Whether isolated loss of septal conduction with preserved BB integrity produces a recognizable electrocardiographic signature remains uncertain. The limited sample size of our study did not allow identification of consistent atypical P-wave morphologies specifically attributable to the absence of septal conduction. Nevertheless, several non-classical P-wave patterns not fulfilling established IAB criteria were observed, underscoring the complexity of interatrial conduction in this population and suggesting that current ECG definitions may not fully capture its spectrum.

Percutaneous closure of large ostium secundum ASDs poses specific technical challenges and may be achieved using either non-self-centering or self-centering devices. Non-self-centering devices primarily act by defect coverage and are associated with limited electromechanical interaction, whereas self-centering devices rely on a stenting mechanism that allows closure of larger defects but may exert greater mechanical stress on atrial structures, with a theoretical impact on atrial and atrioventricular conduction [36,37,38]. The GORE^®^ Cardioform ASD Occluder, owing to its softness and conformability, is particularly attractive in pediatric patients and has been associated with favorable geometric and electrical remodeling, including reductions in P-wave dispersion and arrhythmic risk related to right-sided volume overload [27,28,29,30,31,39].

In our study, new-onset first-degree IAB was observed in a minority of patients shortly after device implantation; however, overall IAB prevalence did not differ significantly before and after closure. No patient with pre-existing IAB exhibited progression to a more advanced conduction abnormality, and in one case, partial IAB resolved following the procedure. Moreover, IAB occurrence was unrelated to device type, and no significant changes in P-wave morphology or time-domain parameters were detected after closure. Collectively, these findings argue against a relevant procedural or device-related effect on interatrial conduction and instead support the hypothesis of a congenital conduction abnormality independent of both hemodynamic load and interventional treatment.

Traditionally, the increased arrhythmic burden observed in patients with ASD has been attributed mainly to chronic right-sided volume overload and its associated structural and electrical remodeling. Our results suggest that IAB may reflect a possible intrinsic conduction vulnerability that appears not strictly related to defect size or hemodynamic burden in this cohort; however, given the limited sample size, these findings should be considered hypothesis-generating and require confirmation in larger studies. While percutaneous ASD closure effectively reverses overload-related remodeling, it does not appear to modify this intrinsic conduction abnormality. Consequently, patients with IAB may remain at increased arrhythmic risk despite successful correction of the defect.

From a clinical perspective, careful analysis of P-wave morphology on standard ECGs represents a simple, noninvasive, and widely available tool for identifying subtle interatrial conduction abnormalities with potential long-term prognostic relevance. Pediatric patients may derive particular benefit from early recognition of IAB, as arrhythmic manifestations often emerge years or decades after defect closure. The relatively short follow-up period precludes any definitive conclusions regarding long-term arrhythmic risk. The absence of early arrhythmic events should not be interpreted as evidence of long-term safety, and extended longitudinal follow-up is required to assess potential late conduction disturbances.

Several limitations of this study should be acknowledged. First, although data were collected prospectively in a structured institutional database, the present analysis was retrospective and single-center in nature, which may limit the generalizability of the findings. As our institution serves as a national referral center with extensive expertise in pediatric and congenital cardiac interventions, a disproportionate number of patients with complex or challenging anatomical features are routinely referred, potentially introducing selection bias. The study population was limited to patients with ostium secundum ASD undergoing percutaneous closure. Patients treated surgically and those with other ASD subtypes (such as sinus venosus or ostium primum defects) were not included. Therefore, the present findings cannot be generalized to surgical populations or to other anatomical variants of atrial septal defect.

Given the limited sample size and number of IAB events, the study may have been underpowered to detect moderate associations between anatomical parameters, including ASD size, and interatrial block, and the possibility of Type II error cannot be excluded.

Second, the analysis was intentionally restricted to the acute post-procedural phase (within 24 h of device implantation). This choice reflects the structure of patient follow-up, which for most individuals is conducted at the referring institutions rather than at our center. Nonetheless, the focus on the immediate post-procedural period was appropriate for the primary objective of the study, as it allowed evaluation of whether interatrial block occurrence was influenced by the closure procedure itself. The long-term persistence or reversibility of newly observed first-degree interatrial block after percutaneous ASD closure could not be assessed and would require serial electrocardiographic evaluations over extended follow-up.

Third, hemodynamic parameters were prioritized over echocardiographic measurements to better address the specific electrophysiological aims of the investigation. While this approach was methodologically justified, it precluded a more detailed analysis of structural atrial remodeling and its potential relationship with interatrial conduction abnormalities.

Finally, the follow-up duration was relatively limited. Given that atrial arrhythmias in pediatric patients with congenital heart disease may develop years or even decades after intervention, the present study was not designed to evaluate the direct impact of interatrial block on long-term arrhythmic outcomes. Nevertheless, the association between interatrial block and increased arrhythmic risk has been consistently demonstrated in previous studies, supporting the clinical relevance of our findings despite the absence of long-term outcome data. Furthermore, the diagnostic criteria for interatrial block were derived from adult populations. Although no pediatric-specific thresholds have been validated, the use of adult-based criteria may have influenced prevalence estimates in younger patients. Future studies should address the need for age-adjusted reference values for interatrial conduction assessment in pediatric populations.

## 5. Conclusions

In this exploratory single-center cohort of pediatric patients with large ostium secundum ASD, interatrial block was observed in approximately one-quarter of cases. Its occurrence was not significantly associated with defect size or hemodynamic parameters but was related to anthropometric variables at the time of closure, suggesting that IAB may become more apparent with increasing age and somatic growth, although the potential influence of timing of repair could not be formally assessed in this cohort. These findings should be interpreted with caution, given the limited sample size. No early arrhythmic events were documented; however, the short duration of follow-up does not allow conclusions regarding long-term electrophysiological outcomes. Although percutaneous ASD closure corrects the hemodynamic burden, its effect on interatrial conduction patterns remains uncertain. The clinical significance of interatrial block in this population requires confirmation in larger, longitudinal studies.

## Figures and Tables

**Table 1:** Baseline characteristics.

	All (n = 37)	IAB (n = 9)	No IAB (n = 28)	*p*-Value
Age (y)	6 (5–11)	12 (5–14)	6 (4–9)	0.073
Weight (kg)	23 (17–47)	46 (24–56)	21 (16–28)	**0.017**
Height (cm)	119 (107–148)	159 (123–169)	115 (106–129)	**0.019**
BSA (m^2^)	0.9 (0.7–1.4)	1.4 (0.9–1.6)	0.8 (0.7–1.0)	**0.024**
Qp/Qs	1.69 (1.32– 2.24)	1.61 (1.40–2.32)	1.79 (1.30–2.24)	0.896
Qp/Qs > 2 (%)	16 (43.2)	3 (33.3)	13 (46.4)	0.654
mPAP (mmHg)	19 (17–22)	16 (14–23)	19 (18–22)	0.221
mPAP > 20 mmHg (%)	15 (40.5)	3 (33.3)	12 (42.9)	0.727
ASD diameter (mm)	20 (18–22)	20 (19–32)	20 (18–22)	0.399
ASD diameter/weight (mm/kg)	0.8 (0.7–1.1)	0.7 (0.4–0.8)	0.9 (0.8–1.2)	**0.027**
ASD diameter/weight > 1.2 mm/kg (%)	9 (24.3)	1 (11.1)	9 (32.1)	0.064
ASD diameter/BSA (mm/m^2^)	23.1 (19.1–27.5)	19.0 (16.8–19.1)	23.3 (19.9–27.8)	0.354
ASD diameter/BSA > 20 mm/m^2^ (%)	27 (73.0)	4 (44.4)	23 (82.1)	0.064
Multi-fenestrated septum	6 (16.2)	3 (33.3)	3 (10.7)	0.073
Fluoroscopy time (min)	9.4 (8.3–15.2)	11.2 (7.7–16.1)	9.4 (8.4–15.2)	0.896
Procedural time (min)	60 ± 30	61 ± 30	60 ± 30	0.980
ASD closure device GCO others	25 (67.6) 12 (32.4)	4 (44.4) 5 (55.6)	21 (75.0) 7 (25.0)	0.088

**Table 2:** Electrocardiographic findings.

	Pre-Procedural Data (n = 37)	Post-Procedural Data (n = 37)	*p*-Value
Heart rate (bpm)	96 ± 16	93 ± 16	0.829
Total P-wave duration (mm)	100 (100–120)	110 (100–120)	0.814
Maximum P-wave duration (mm)	100 (90–115)	100 (90–110)	1.000
Minimum P-wave duration (mm)	60 (60–75)	60 (50–80)	1.000
Isoelectric interval (mm)	0 (0–10)	10 (0–10)	0.619
P-wave dispersion (mm)	40 (30–50)	40 (25–50)	1.000
P-wave axis	45 (30–60)	45 (30–60)	1.000
Maximum P-wave amplitude	0.18 (0.15–0.20)	0.15 (0.15–0.20)	0.810
Minimum P-wave amplitude	0.00 (0.00–0.05)	0.05 (0.00–0.05)	0.619
P-wave voltage in lead I	0.10 (0.10–0.15)	0.10 (0.10–0.15)	1.000
P-wave area	7.2 ± 2.5	7.2 ± 2.6	1.000
P-wave terminal force in V1	−20 (−40–−5)	−20 (−40–−10)	1.000
PR duration	140 (130–155)	140 (130–140)	0.813

**Table 3:** Interatrial block occurrence pre- and post-procedure.

	Pre-Procedural (n = 37)	Post-Procedural (n = 37)	*p*-Value
No IAB (%)	28 (75.7)	26 (70.2)	0.628
IAB First degree (%) Second degree (%) Third degree (%)	8 (21.6) 0 (0) 1 (2.7)	10 (27.0) 0 (0) 1 (2.7)	0.597
Any IAB (%)	9 (24.3)	11 (29.7)	0.734

**Table 4:** Univariate and bivariate logistic regression for interatrial block.

	Univariate		Bivariate	
	OR (95% CI)	*p*-Value	OR (95% CI)	*p*-Value
Age	1.20 (0.99–1.45)	0.059		
Weight (kg)	1.06 (1.01–1.11)	**0.032**		
Height (cm)	1.04 (1.01–1.08)	**0.017**		
BSA (m^2^)	13.65 (1.44–129.50)	**0.023**	9.86 (0.90–107.92)	0.061
Qp/Qs	1.20 (0.40–3.58)	0.750		
mPAP	0.87 (0.70–1.08)	0.209		
ASD diameter	1.15 (0.98–1.37)	0.082	1.09 (0.92–1.28)	0.312
GCO closure device	0.44 (0.10–1.92)	0.276		

## Data Availability

The data underlying this article will be shared upon reasonable request to the corresponding author.
